# NapM enhances the survival of *Mycobacterium tuberculosis* under stress and in macrophages

**DOI:** 10.1038/s42003-019-0314-9

**Published:** 2019-02-15

**Authors:** Yu Liu, Zhiwei Xie, Xiling Zhou, Weihui Li, Hua Zhang, Zheng-Guo He

**Affiliations:** College of Life Science and Technology, Huazhong Agricultural University, Wuhan, 430070 China

## Abstract

Hostile environmental cues cause *Mycobacterium tuberculosis* to enter a state of slow growth for survival. However, the underlying regulatory mechanism remains unclear. DnaA is essential for DNA replication initiation and represents an efficient target for growth regulation in bacteria. Here, we show that the nucleoid-associated protein NapM is a DnaA antagonist, protecting *M. tuberculosis* from stress-mediated killing. NapM can be induced by diverse stressful signals. It binds to DnaA to inhibit both its DNA replication origin-binding and ATP hydrolysis activity. As a DnaA antagonist, NapM inhibits the mycobacterial DNA synthesis in vitro and in vivo in *M. tuberculosis*. Furthermore, we show that NapM contributes to the survival of *M. tuberculosis* under stress and within macrophages during infection. Our findings provide a previously unidentified mechanism of mycobacterial survival under stress and also suggest NapM as a potential drug target for tuberculosis control.

## Abstract (web summary)

Yu Liu, Zhiwei Xie et al. show that nucleoid-associated protein NapM enhances the survival of *M. tuberculosis* in macrophages under stress by inhibiting DNA synthesis as a DnaA antagonist. This study suggests NapM as a potential drug target for tuberculosis control.

## Introduction

Most bacteria can survive under stressful conditions^1–3^. This is an important strategy for a pathogen to escape from the killing mechanisms of a host and achieve successful infection^4^. *Mycobacterium tuberculosis*, the causative agent of tuberculosis, is a notorious intracellular pathogen that causes millions of global deaths annually^5^. *M. tuberculosis* has unique abilities to survive in the host and to cope with the pressure of the host environment, which leads to difficulty in controlling the infection of tuberculosis^6,7^. However, the molecular mechanism underlying such stress-inducible survival remains largely unclear.

Recent studies show that bacteria have evolved mechanisms to transduce environmental cues into the cell-cycle engine and can thus reprogram their growth to rapidly adapt to stress conditions^8^. The growth and replication of nearly all bacteria essentially depend on the conserved ATPase protein DnaA, which binds to the replication origin (ori) and unwinds DNA^9^. By contrast, excess DnaA in bacterial cells results in replication over-initiation and subsequent growth inhibition^10^. Therefore, DnaA is an efficient target for growth regulation during the cell cycle, and the amount and activity of DnaA must be strictly controlled^11^. Notably, *M. tuberculosis* enters a state of very slow replication when encountering a hostile environment, and this strategy contributes to its survival within the host^12^. The regulation of DnaA expression has recently been found to be associated with the drug-inducible survival of *M. tuberculosis*^13^. Nevertheless, very little is known regarding the strategy of DnaA activity regulation and its relationship with the stress-inducible survival of *M. tuberculosis*.

Our group has characterized a conserved protein in mycobacteria, NapM (Rv0047c), as a nucleoid-associated protein^14^. In the present study, we have shown that NapM acts as a broad stress-inducible factor. It acts as a DnaA antagonist in *M. tuberculosis* and is required for the pathogen’s survival under stress. Our findings provided new clues for understanding the regulatory mechanism of pathogenesis in this important human pathogen.

## Results

### *M. tuberculosis* NapM is stress-inducible

**Fig. 1:**
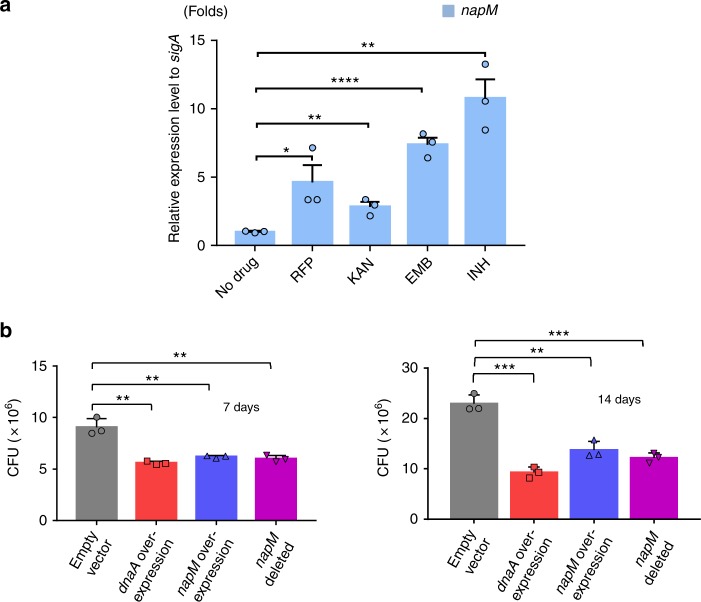
*M. tuberculosis napM* is induced by various antibiotics and affects mycobacterial growth. **a** *napM* was induced by various anti-TB antibiotics in *M. tuberculosis*. qRT-PCR assays were used to determine the relative expression level of *napM* in *M. tuberculosis* upon induction by various anti-TB antibiotics. *M. tuberculosis* was exposed to rifampicin, kanamycin, ethambutol, and isoniazid. *napM* expression was normalized by invariant transcript *sigA* gene. Bars indicate the means ± standard errors calculated from three independent biological experiments. The *P* values of the results (<0.05, <0.01, and <0.001) are indicated by asterisks (*), double asterisks (**), and triple asterisks (***), respectively. Two-tailed *P* values are RFP 0.0279, KAN 0.0024, EMB 0.0001, and INH 0.0062 when compared to the no-drug control. **b** Both *napM* deletion and *napM* overexpression inhibited the growth of *M. tuberculosis* H37Ra. Bacterial counts were determined at two representative time points, 7 and 14 days. Symbols represent each biological replicate and bars indicate means ± standard errors calculated from three independent biological experiments. The *P* values of the results (<0.05, <0.01, and <0.001) are indicated by asterisks (*), double asterisks (**), and triple asterisks (***), respectively. Two-tailed *P* values are 0.0022 for dnaA overexpression, 0.0043 for napM overexpression, and 0.0041 for napM-deleted at 7 days, and *P* values are 0.0003 for dnaA overexpression, 0.0028 for napM overexpression, and 0.0007 for napM-deleted at 14 days when compared to the control empty vector. qRT-PCR, quantitative real-time PCR

This work was prompted by an observation that *napM* expression is correlated with stress. As shown in Fig. 1a, *M. tuberculosis* H37Ra exposure to several major anti-TB drugs increased *napM* expression three- to sixfold compared with drug–stress absence. By contrast, a similar expression change of the negative control *sigA* was not observed under the same conditions. Interestingly, a search of previously published data and the TB database (http://www.tbdb.org/) produced by other groups revealed that *napM* expression in several mycobacterial species was induced almost more than twofold by different drugs and several stressful growth conditions (Supplementary Table 1). Quantitative real-time PCR (qRT-PCR) assays further demonstrated *napM* induction when *M. tuberculosis* H37Ra was exposed to additional conditions, such as heat shock, oxidative stress, acid shock, cell-membrane damage, and nutrition starvation (Supplementary Fig. 1). Therefore, NapM can be induced by diverse stressful signals and can be a broad stress-inducible protein in *M. tuberculosis*.

### NapM affects *M. tuberculosis* growth

To further investigate the effect of *napM* on *M. tuberculosis* growth, an *napM*-deleted *M. tuberculosis* H37Ra mutant strain was initially generated using gene-replacement strategy. As shown in Fig. 1b, *napM* deletion resulted in growth inhibition compared with the wild-type strain, and this inhibition was more obvious with prolonged culture time from 7 to 14 days. Obvious growth inhibition was also observed for the *napM* overexpression strain, which was very similar to the case of the *napM* deletion strain (Fig. 1b). These results indicated that the level of NapM expression affected mycobacterial growth, and both excessive and insufficient NapM inhibited the growth of *M. tuberculosis*. The essential DNA replication initiation gene *dnaA* had a similar effect on mycobacterial growth, and *dnaA* overexpression obviously inhibited *M. tuberculosis* growth (Fig. 1b), consistent with previous reports^10^. Most interestingly, the coexpression of *napM* and *dnaA* neutralized their own inhibition, and the recombinant strain grew similarly as the wild-type strain (Supplementary Fig. 2).

### NapM physically interacts with DnaA

**Fig. 2:**
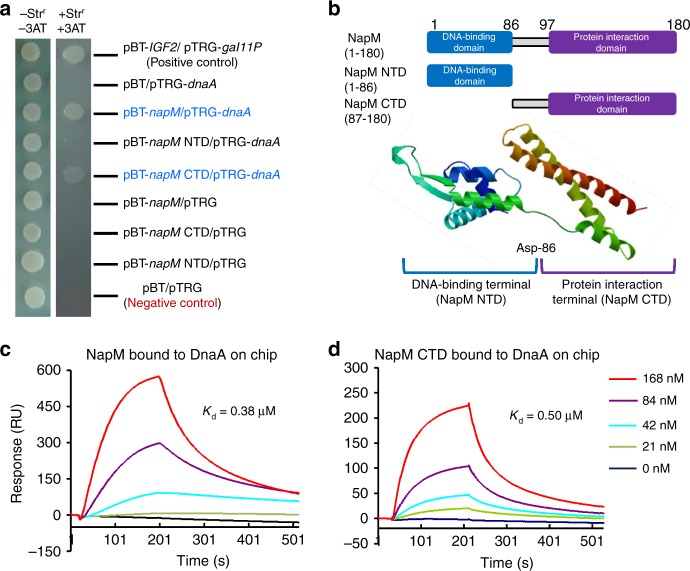
NapM physically interacts with DnaA. **a** Bacterial two-hybrid assays. *Escherichia coli* reporter strains with various recombinant plasmids were spotted on the plate with or without streptomycin (str) and 3-amino-1,2,4-triazole (3-AT). **b** Diagram of NapM and two of its truncated derivatives. The three-dimensional structure of NapM was predicted by SWISS-MODEL. **c**, **d** Surface plasmon resonance assays. Different amounts of NapM (**c**) or NapM CTD (**d**) were passed over the CM5 chip immobilized by 5.7 μM of DnaA. Overlay plots depicting the interactions were then produced. The dissociation constant (*K*_d_) for each interaction was indicated. CTD, C-terminal domain

NapM and DnaA can mutually neutralize their own inhibition on the growth of *M. tuberculosis*, suggesting an interaction between these two proteins. Accordingly, we examined their interaction using bacterial two-hybrid assays. As shown in Fig. 2a, the co-transformants containing *napM*/*dnaA* grew well on the screening medium. By contrast, the negative and self-activation controls did not grow under the same conditions. To further map the potential region in NapM responsible for this interaction, two mutants, the N-terminal DNA-binding domain and C-terminal domain (CTD), were constructed (Fig. 2b). The co-transformants containing *napM* CTD/*dnaA* grew similarly as the co-transformants *napM*/*dnaA* on the screening medium, indicating that NapM interacted with DnaA through its CTD.

A surface plasmon resonance (SPR) assay confirmed the physical interaction between NapM and DnaA. DnaA protein was initially immobilized on the surface of CM5 chip. Response increased with increased NapM protein (21, 42, 84, and 168 nM) amounts passed over the chip (Fig. 2c), indicating that the purified NapM protein can interact with DnaA in vitro. Furthermore, the dissociation constant (*K*_d_) for the specific interaction between NapM and DnaA was measured to be 0.38 μM, indicating a strong binding affinity between NapM and DnaA. No response was obtained when the negative control protein GroEL 2 was passed over the chip (Supplementary Fig. 3). The NapM CTD mutant protein was shown to retain a clear interaction with DnaA with a *K*_d_ of 0.5 μM (Fig. 2d).

A co-immunoprecipitation (Co-IP) experiment further confirmed the interaction of these two proteins in mycobacteria in vivo. Protein A beads that were conjugated with an antibody raised against NapM were used for our Co-IP assays. As shown in Supplementary Fig. 4, DnaA was clearly associated with NapM because an obvious and specific hybridization signal was detected (lane 2). The size of the signal band matched the size of purified DnaA (lane 4) and cell extraction of DnaA (lane 1), and no signal was detected for the negative control sample (lane 3), in which Protein A beads were not conjugated to the anti-NapM antibody. Collectively, these results suggested that NapM physically interacted with DnaA.

### NapM inhibits the binding of DnaA to *oriC* DNA

**Fig. 3:**
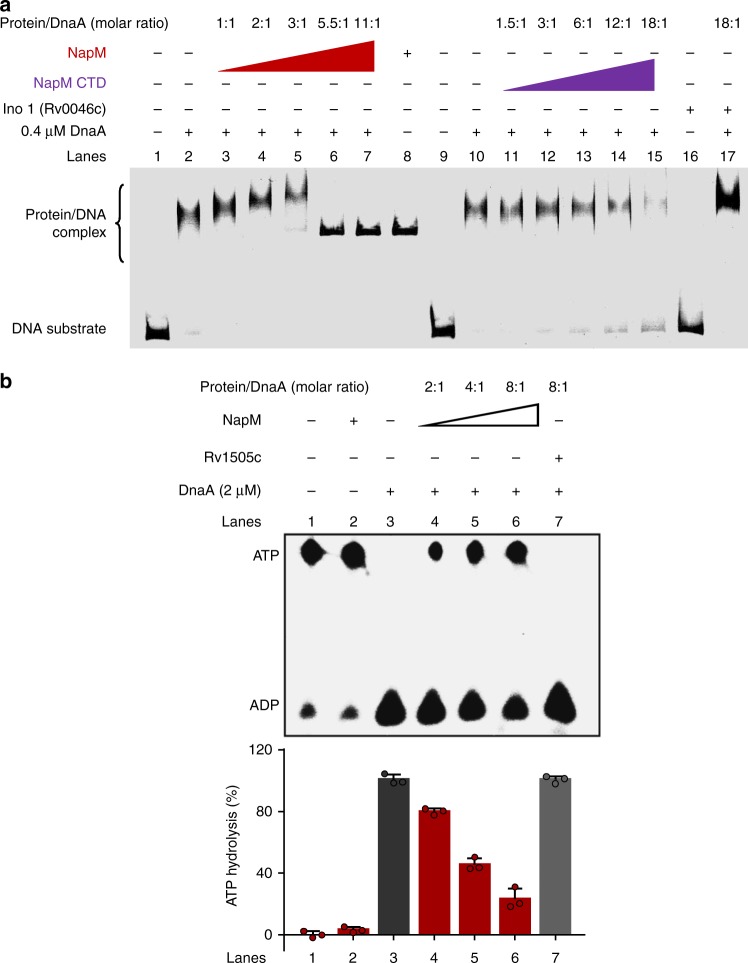
NapM inhibits both the DNA-binding activity and ATPase activity of DnaA. **a** EMSA assays for the inhibition of the DNA-binding activity of DnaA. Fluorescent-labeled *oriC* was co-incubated with DnaA protein in the absence or presence of NapM, NapM CTD, or the negative control protein Ino 1 (Rv0046c). **b** TLC assays for the inhibition of ATPase activity of DnaA. A fixed amount of DnaA was used to degrade [α-^32^P] ATP in the presence of different concentrations of NapM (4–16 μM). The phosphate released in ATPase assay was quantified using Typhoon Variable Mode Imager, and the mean values of three independent biological experiments are shown in the lower panel. CTD, C-terminal domain; EMSA, electrophoretic mobility shift assay; TLC, thin-layer chromatography

Direct interaction between NapM and DnaA suggested that NapM was involved in the regulation of DnaA activity. To test this hypothesis, we first assayed the effect of NapM on the DNA origin-binding activity of DnaA, which is one of the key steps in DNA replication initiation. As shown in Fig. 3a, our electrophoretic mobility shift assays (EMSAs) showed that either DnaA or NapM alone bound with *oriC* and that the complex band of NapM-*oriC* was a little lower than that of DnaA-*oriC* on the gel (lanes 2 and 8). A larger protein–DNA complex (NapM–DnaA–*oriC* DNA complex) appeared on the gel with an increased amount of NapM protein added into the reaction mixture containing fixed concentrations of DnaA and DNA. However, with progressively increased ratio of NapM/DnaA from 1:1 to 3:1 (lanes 3–5), the amount of protein/DNA complex decreased. Meanwhile, a NapM–DNA complex began to appear on the gel when the NapM/DnaA mixture was of 3:1 ratio, and the DnaA–*oriC* complex band almost disappeared at 5.5:1 ratio, indicating that DnaA–*oriC* complex formation competed with NapM protein. Interestingly, if NapM CTD was used to replace NapM to conduct a similar experiment as above, DnaA/*oriC* complex formation was also inhibited although a relatively higher concentration of NapM CTD was required in the reactions (NapM CTD/DnaA ratio of 3:1–18:1); however, no larger protein–DNA complex was clearly observed. This finding was most likely due to the fact that NapM CTD-DNA retained only partial interaction ability, which was insufficient to form a stable complex with DnaA/*oriC* on the gel under our experimental conditions. Moreover, NapM CTD lost its DNA-binding ability, and no additional NapM CTD -DNA band was observed (Supplementary Fig. 5a), indicating that NapM inhibited the DNA-binding activity of DnaA through direct protein–protein interaction but not through DNA-binding competition. DnaA also had an obviously stronger DNA-binding activity (*K*_d_, 60.5 nM) than NapM (*K*_d_, 458 nM) (Supplementary Fig. 6).

Taken together, our data suggested that NapM inhibited the *oriC-*binding activity of DnaA through physical interaction with the protein.

### NapM inhibits the ATPase activity of DnaA

ATPase activity promoted rapid oligomerization of DnaA on *oriC* in *M. tuberculosis*^15^. We hypothesize that NapM affects the ATPase activity of DnaA negatively and thus inhibits the rapid oligomerization of DnaA through protein–protein interaction. To test this theory, we used thin-layer chromatography (TLC) assays to determine the ATPase activity of DnaA, both in the presence and in the absence of NapM protein. As shown in Fig. 3b, increased concentrations of NapM (lanes 4–6) added into the reactions progressively suppressed the ATPase activity of DnaA. By contrast, ATPase activity was unaffected when the negative control protein Rv1505c was added into similar reactions (lane 7). Either NapM (lane 2) or Rv1505c (Supplementary Fig. 5b) alone had no ATPase activity. These results suggested that NapM physically interacted with DnaA to specifically inhibit its ATPase activity.

### NapM inhibits in vitro DNA synthesis

**Fig. 4:**
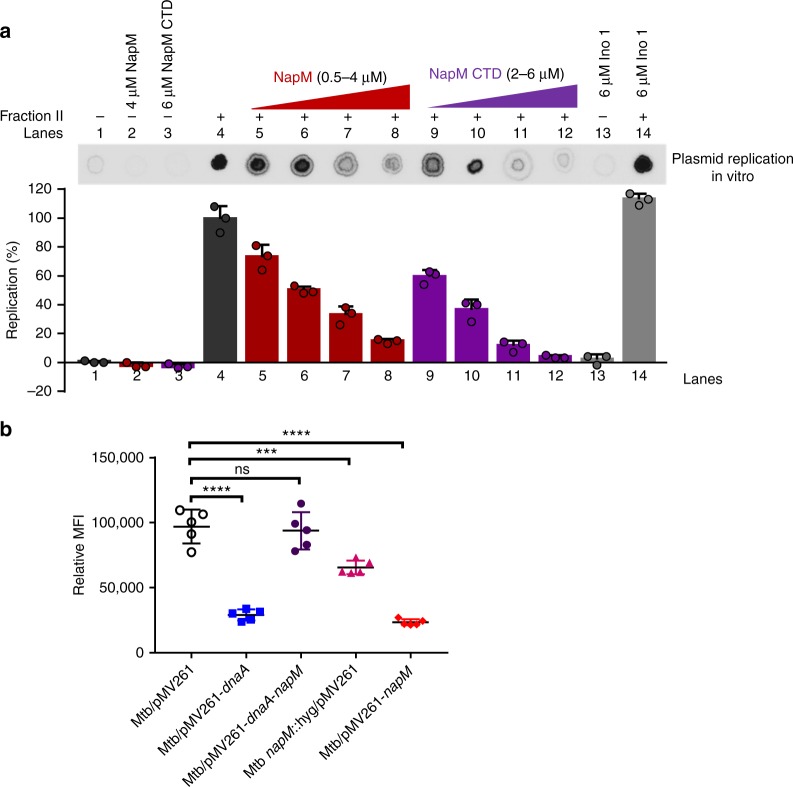
NapM affects DNA replications both in vitro and in vivo. **a** NapM inhibits DNA synthesis in vitro. DNA replication was carried out with ammonium sulfate-fractionated crude *M. tuberculosis* cell lysate (fraction II) in the presence of increasing concentrations of purified NapM or NapM CTD. As a control, pUC-*oriC*_*Mtb*_ was either loaded alone or with NapM (lane 2), NapM CTD (lane 3), and Ino 1 (lane 13), respectively. The replication products were purified and blotted on a nylon membrane and then quantified using Typhoon Variable Mode Imager. Representative DNA synthesis bound on a nylon membrane is indicated on top of the panel. Bars indicate means ± standard errors calculated from three independent biological experiments. **b** NapM affects DNA replication in vivo in *M. tuberculosis*. Mean fluorescence intensity (MFI) of DAPI-stained samples relative to an unstained sample was determined by flow cytometry. Representative histograms of flow cytometry are found in Supplementary Fig. 7. Symbols represent each biological replicate and bars represent means ± standard errors calculated from five independent biological experiments. Asterisk represents the significant difference (****P < 0.0001, ***P < 0.001; two-tailed Student’s *t* test) between two groups. Two-tailed *P* values are 0.0001 for Mtb/pMV261-*dnaA*, 0.9502 for Mtb/pMV261-*dnaA*-*napM*, 0.0002 for Mtb *napM*::/pMV261, and 0.0001 for Mtb/pMV261-*napM* when compared to control Mtb/pMV261. CTD, C-terminal domain; DAPI, 4,6-diamidino-2-phenylindole

NapM inhibited both the ATPase activity of DnaA and its binding to *oriC* DNA, both of which are essential for DNA replication initiation^16,17^, suggesting that NapM suppressed DNA replication. For confirmation, we conducted an in vitro DNA replication assay. The fraction with DNA replication activity was produced from *M. tuberculosis* H37Ra. With increased concentrations of NapM (Fig. 4a, lanes 5–8) added into the fractions and after incubation at 30 °C for 90 min, the amount of total nucleotide incorporation decreased, indicating that NapM inhibited the DNA synthesis of the mycobacterial cell fraction in vitro. NapM CTD, which retained partial interaction ability with DnaA but lost DNA-binding activity, presented a slightly similar inhibition on nucleotide synthesis (Fig. 4a, lanes 9–12). By contrast, no effect was observed for the nucleotide incorporation activity of the fraction when a negative control protein Ino 1 (Rv0046c) was added into similar reactions (lane 14). Therefore, these results suggested that NapM inhibited the in vitro DNA replication activity of *M. tuberculosis* fraction.

### NapM affects DNA replication in vivo

To further examine if NapM also affects DNA replication in vivo in *M. tuberculosis*, we utilized flow cytometry to measure the DNA content per cell of the wild-type *M. tuberculosis* strain and its various mutant strains. As shown in Fig. 4b and Supplementary Fig. 7, compared with the wild-type mycobacterial cells (Mtb/pMV261), *napM*-overexpressed *M. tuberculosis* (Mtb/pMV261-*napM*) obtained a much lower DNA content based on the determination of mean fluorescence intensity of 4,6-diamidino-2-phenylindole staining, the result of which is very similar to the observation for *dnaA*-overexpressed strain (Mtb/pMV261-*dnaA*). In addition, the *napM*-deleted mutant (Mtb *napM*::/pMV261) also obtained a lower DNA content although to a lesser extent when compared with the wild-type strain (Fig. 4b). These results indicate that NapM affects DNA replication in vivo. Strikingly, coexpression of *napM* and *dnaA* in *M. tuberculosis* (Mtb/pMV261-*dnaA*-*napM*) could neutralize either of their respective inhibitions on DNA synthesis, and the DNA content of the coexpression strain was similar to that of the wild-type strain (Fig. 4b). This suggests that NapM inhibits the activity of DnaA on DNA replication, which is exactly consistent with the results above (Figs. 2, 3, and 4a).

Therefore, our results indicated that NapM affects DNA replication in vivo, and the physical interaction of NapM with DnaA inhibits the function of DnaA in vivo in *M. tuberculosis*. NapM has a brake-like role on regulating the activity of DnaA, and either excess or deletion of NapM inhibits DNA synthesis.

### NapM is vital for stress-inducible survival

**Fig. 5:**
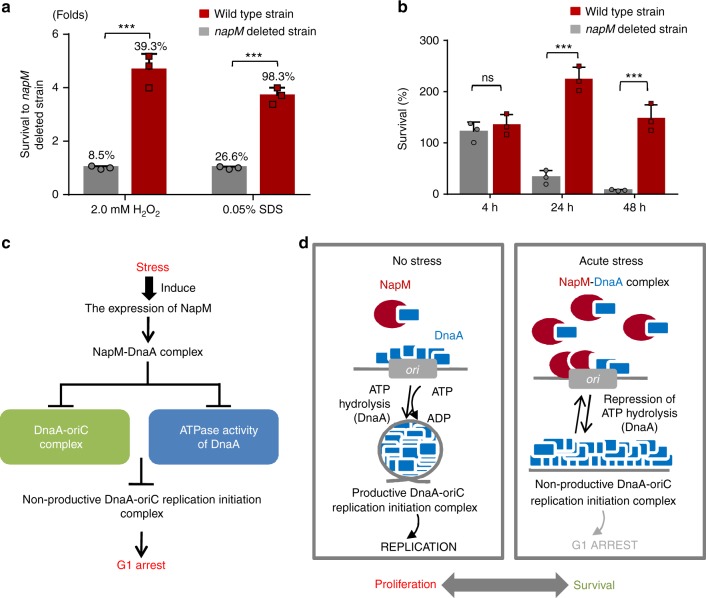
NapM is essential for the survival of *M. tuberculosis* under stress. NapM was required for the survival of *M. tuberculosis* under H_2_O_2_ and SDS stress (**a**) and within macrophages during infection (**b**). RAW264.7 cells were infected with mutant or wild-type strains of *M. tuberculosis* H37Ra, and the mycobacterial survival was assayed by determining colony-forming units (CFUs) at 4, 24, and 48 h after infection. Symbols represent each biological replicate and bars indicate means ± standard errors calculated from three independent experiments. The *P* values of the results (<0.001) are indicated by triple asterisks (***). **c, d** A summary for the NapM-induced regulation of DNA replication initiation and *M. tuberculosis* growth. A pathway describing DnaA activities regulated by NapM (**c**). A model showing that NapM coupled environmental signals with the regulation of DNA replication initiation to promote either proliferative or survival mode in *M. tuberculosis* (**d**). SDS, sodium dodecyl sulfate

NapM inhibited DNA replication initiation, and its expression coupled with multiple stressful inductions, suggesting that this protein may contribute to the stress-inducible growth of mycobacteria. Accordingly, we examined the effects of NapM on mycobacterial survival under stressful conditions, such as oxidative stress and cell-membrane damage stress. As shown in Fig. 5a, we observed different colony-forming abilities between wild-type and *napM-*deleted *M. tuberculosis* H37Ra strains upon exposure to 2.0 mM H_2_O_2_ for 24 h. After the stress was removed, mycobacterial colonies were counted. The colony-forming unit (CFU) of wild-type *M. tuberculosis* H37Ra was about 4.6-fold higher than that of the *napM-*deleted mutant strain. We also observed a similar effect of NapM, although a little less, on mycobacterial survival when bacterial cells were exposed to cell-membrane damage stress (by sodium dodecyl sulfate (SDS)) (Fig. 5a). These results indicated that NapM contributed to the survival of *M. tuberculosis* under stressful conditions.

During infection, *M. tuberculosis* is exposed to acute intracellular stresses. To further assess the effect of NapM on the intracellular survival of *M. tuberculosis*, macrophage RAW264.7 was used as a model and was infected with wild-type or mutant mycobacterial strains. As shown in Fig. 5b, wild-type *M. tuberculosis* was demonstrated to survive well within the macrophages for 48 h. By contrast, the *napM*-deleted mutant strain retained a much lower survival percentage (10% at 24 h and 3% at 48 h after infection) than the wild-type *M. tuberculosis* strain, indicating that NapM played an essential role in *M. tuberculosis* survival within macrophages.

## Discussion

NapM has recently been characterized as a nucleoid-associated protein and is conserved in all sequenced mycobacterial genomes^14^. In the present study, we have shown that NapM was a broad stress-inducible protein in *M. tuberculosis* and can regulate mycobacterial growth. NapM physically interacted with DnaA and inhibited DNA replication initiation both in vitro and in vivo. Strikingly, the protein was shown to contribute to the survival of *M. tuberculosis* under stressful conditions and within macrophages during infection. In this work, we reported a novel regulatory mechanism on the survival of *M. tuberculosis* under stress.

To ensure that chromosomal DNA is accurately replicated and that this process is correlated with cell growth, bacteria must tightly regulate the initiation step of DNA synthesis, especially under stressful conditions^18^. Many studies suggest that *M. tuberculosis* can retain slow growth and replication under environmental stresses^1,2^. However, the underlying regulatory mechanisms remain unclear. The regulation of DNA replication initiation can efficiently prevent over-replication or decrease bacterial growth under stressful environments^19^. One of our most interesting findings was that NapM can deliver environmental cues into the DNA-replication engine and can thus potentially influence the growth of *M. tuberculosis* under stress. NapM directly targeted DnaA to prevent its binding to *oriC*, which decreased the amount of DnaA in *oriC*. Meanwhile, NapM inhibited the ATPase activity of DnaA, which suppressed its rapid oligomerization in *oriC*. Several proteins from different bacteria have been identified as a direct DnaA-binding factor, such as DiaA in *Escherichia coli*, HobA in *Helicobacter pylori*, and YabA in *Bacillus subtilis*^20–22^. However, NapM did not share sequence identity with these regulators. NapM was also a broad stress-inducible protein and was essential to the survival of *M. tuberculosis* under stress. Therefore, NapM was a novel DnaA-interacting protein.

Our findings supported a model in which, upon stress induction, NapM inhibited DnaA activity in regulating the growth and replication of *M. tuberculosis* (Fig. 5c). We propose that the absence of stress induction relatively limited NapM expression, thereby enabling the stable accumulation of DnaA in *M. tuberculosis* for normal DNA replication initiation and bacterial growth. However, hostile environments induce the rapid expression of NapM. Excessive NapM can prevent DnaA from binding with *oriC* and inhibit its ATPase activity, both of which can suppress the formation of the productive DnaA–*oriC* replication–initiation complex. Therefore, DNA replication initiation can be sequestered and *M. tuberculosis* can retain a slow growth for survival under environmental stresses.

As an important pathogen in humans, *M. tuberculosis* has a striking ability to survive in hostile environments during infection^23,24^. This ability explains the difficulty in controlling tuberculosis. Our most interesting finding was the crucial role of NapM in the survival of *M. tuberculosis* within macrophages during infection. NapM can couple environmental signals with the regulation of DNA replication initiation to protect *M. tuberculosis* against environmental stresses for survival. Thus, a novel target for killing dormant bacteria and a potential strategy for tuberculosis control were suggested.

## Methods

### Plasmids, enzymes, and chemicals

pET28a (Novagen) was used for the overexpression of *M. tuberculosis* proteins in the *E. coli* strain BL21 (λDE3) (Novagen, Darmstadt, Germany). All antibiotics and enzymes, including T4 ligase, DNA polymerase, and restriction enzymes, were obtained from TaKaRa Biotech (Shiga, Japan). Ni–NTA (Ni^2+^–nitrilotriacetate) agarose columns were purchased from Qiagen (Hilden, Germany). Invitrogen (Carlsbad, USA) synthesized all PCR primers for our assay. The radioactive-labeled [α-^32^P]ATP was purchased from Perkin Elmer (USA). Middlebrook 7H9 medium and Oleic acid-Albumin-Dextrose-Catalase (OADC) enrichment were purchased from Becton-Dickinson Company (USA) and were used for growing *M. tuberculosis*.

### Cloning, expression, and purification of NapM and DnaA

A pair of primers (5′-AGACGAATTCAAATGCTGGAGCTCGCCAT-3′ and 5′-ACAGTCTAGATTACGTCTGTTCGGCGGG-3′) was used to amplify the *napM* gene from *M. tuberculosis* H37Ra genome by PCR. The amplification was performed as follows: 95 °C for 5 min, followed by 30 cycles of 95 °C for 30 s, 50 °C for 30 s, 72 °C for 1 min and a final extension of 72 °C for 10 min, respectively. The *napM* N-terminal DNA-binding domain region was amplified by a pair of primers (5′-AGACGAATTCAAATGCTGG AGCTCGCCAT-3′ and 5′-AGACTCTAGATTAGAAACCGTCGTCGGTGTAG-3′), and the *napM* CTD region was amplified by a pair of primers (5′-ATAT GAATTC AA ATGCCAGCGGGCACCCCGGT-3′ and 5′-ACAGTCTAGATTACGTCTGTTCG GCGGG-3′) from *M. tuberculosis* H37Ra genome. The *dnaA* gene was amplified by using forward primer 5′-ATATGCGGCCGCATTGACCGATGACCCCGG-3′ and reverse primer 5′- GTGGTCTAGACTAGCGCTTGGAGCGCTGAC-3′. The *napM* gene was digested by restriction endonucleases and cloned into modified pET28a for protein overexpression in the host strain *E. coli* BL21 (λDE3). The recombinant proteins were purified according to our previously published procedures^25^. The purity of these proteins was estimated by 15% SDS-polyacrylamide gel electrophoresis (Supplementary Fig. 8c), and its concentrations were determined by spectrophotometric absorbance at 595 nm according to Bio-Rad protein assay.

### Construction of a *napM*-deleted *M. tuberculosis* H37Ra mutant strain

The *napM* gene of *M. tuberculosis* was knocked out according to previously described procedures with some modifications^26^. A pMind-derived suicide plasmid carrying a hygromycin resistance gene was constructed and a *sacB* gene was inserted to confer sensitivity to sucrose as a negative selection marker. pMind-*napM* KO was constructed by inserting the upstream fragment and downstream fragment (amplified with corresponding primers from *M. tuberculosis* H37Ra genome) into the pMind vector. Then, pMind-*napM* KO (1 µg) was electroporated into *M. tuberculosis* H37Ra and selected on 7H10 medium containing 100 µg ml^−1^ hygromycin, 2% sucrose, and 50 µg ml^−1^ X-gal. Both PCR products and the subsequent sequencing analysis (Supplementary Fig. 8a, b) were conducted to prove positive mutants.

### Quantitative real-time PCR assay

Log-phase cultures of the wild-type *M. tuberculosis* strain were diluted 1:10 into 5 ml Middlebrook 7H9 (supplemented with 10% OADC, 0.05% Tween 80, and 0.2% glycerol) medium and was ensured at the same initial OD. Then, the cells were grown under various stressful conditions, including antibiotics (rifampicin, isoniazid, ethambutol, kanamycin), acid shock (pH 4.0), nutrient starvation, heat shock (55 °C), oxidative stress (2.5 μM H_2_O_2_), and cell-membrane damage (0.05% SDS). Nutrient-starvation cultures were set up as previously described^27^ in phosphate buffer saline containing 0.05% Tween 80. Cultures were harvested at an OD_600_ 1.0. Total RNA from *M. tuberculosis* H37Ra was prepared according to previously described procedures^28^. For qRT-PCR, the following primers were used: *napM*, 5′-TCGCCATCCTGGGTCTGT T-3′ (forward) and 5′-CAGCGCCGGGTATAACGAA-3′ (reverse); *dnaA*, 5′-CATC TGACCGCCCACCCAA-3′ (forward) and 5′-AGCCGTTCCATCTGTGCTTTCT-3′ (reverse). For normalizing data, the following *sigA*-specific primers were used: 5′ -CGACGAAGACCACGAAGACC-3′ (forward) and 5′-TTCATCCCAGACGAA ATCACC-3′ (reverse). The real-time PCR was performed in a Bio-Rad CFX RT-PCR machine. The levels of *sigA* or *dnaA* transcripts were used as the internal control to normalize expression levels of different genes, and melting curves were used to analyze amplification specificity. The rates of expression change were calculated by $2^{\Delta \Delta \mathrm{Ct}}$ method. These experiments were performed in triplicate, and error bars indicate standard error.

### Bacterial two-hybrid assay

The BacterioMatch II Two-Hybrid System Library Construction Kit (Stratagene) was used to detect the interactions between DnaA and NapM proteins^29^. Positive-growth co-transformants were characterized on a screening plate containing 5 mM 3-amino-1,2,4-triazole (3-AT) (Stratagene), 8 mg ml^−1^ streptomycin, 15 mg ml^−1^ tetracycline, 34 mg ml^−1^ chloramphenicol, and 50 mg ml^−1^ kanamycin. Co-transformants containing pBT-LGF2 and pTRG-Gal11P (Stratagene) were used as positive controls, and the recombinant strains containing empty vector pBT and pTRG were used as negative controls.

### SPR assay

SPR analysis for the binding of NapM with DnaA was conducted on a Biacore 3000 instrument with CM5 sensor chips according to previously published procedures^30^. Briefly, to detect the physical interaction between NapM (including its N-terminal and C-terminal domains) and DnaA, DnaA protein was immobilized onto the CM5 chips, and then NapM and its mutant proteins were passed over the chips. All proteins were diluted in the reaction buffer (10 mM hydroxyethyl piperazineethanesulfonic acid (HEPES), 150 mM NaCl, 3 mM ethylenediaminetetraacetic acid (EDTA), and 0.01% (vol/vol) surfactant p20) (pH 7.4) to concentrations of 10–128 nM and were injected at 10 ml min^−1^ for 5 min at 25 °C. Each analysis was performed in triplicate. An overlay plot was produced using the BIA evaluation 3.1 software to depict the interaction between NapM and DnaA. In order to identify the interaction between NapM and domains of DnaA, NapM was immobilized onto the NTA chips, and DnaA and its mutant proteins were passed over the chips.

### Co-IP assays

The in vivo interactions in *M. tuberculosis* between NapM and DnaA were analyzed by Co-IP according to previously published procedures with some modifications^29^. Briefly, exponentially growing cells of *M. tuberculosis* H37Ra with recombinant plasmid pMV261-*dnaA* were harvested, resuspended, and lysed in 4 ml of buffer (50 mM Tris-HCl (pH 7.5), 150 mM NaCl, 1 mM EDTA, and 0.5% Nonidet P-40). Co-IPs were performed by incubating and shaking 0.5 mg of the mycobacterial cell extract with 5 μl of NapM antiserum. Finally, the samples were analyzed by western blotting using anti-DnaA antibody. The antibodies were ordered from the Animal Immunology Center of Wuhan Institute of Virology, Chinese Academy of Sciences. Briefly, the purified NapM protein (300–500 µg) was dissolved in 600 µl of Freund’s complete adjuvant and injected into rabbits or mouse. Subsequently, the protein was dissolved in Freund’s incomplete adjuvant for the same immunization after an interval of 14 days. Seven days after the injection, animals were bled for antibody preparation.

### Electrophoretic mobility shift assay

The *oriC* fragments were generated by PCR amplification using a pair of primers (5′-GTGTCGTGAGCTCACCGATC-3′ and 5′-AAAATCTGCCAGCCAGGCC-3′) from H37Ra genome and purified with a DNA purification kit (BioFlux). The 5′-end of DNA substrate was labeled and EMSAs were performed according to previously described procedures^14^. The total volume of the reaction mixtures was 20 μl, including reaction buffer, 1 μM labeled DNA fragments, 0.5 μM DnaA, and various amounts of NapM/ NapM CTD.

### ATP hydrolysis assay

ATPase activity was assayed according to previously described procedures with minor modifications^15^. Specifically, the reaction mixtures (10 μl) contained 20 mM Tris-HCl (pH 8.0), 1 mM MgCl_2_, 100 mM KCl, 8 mM dithiothreitol, 4% sucrose, 80 μg ml^−1^ bovine serum albumin, 1 mM ATP, 3.4 fmol of [α-^32^P] ATP, 1.93 μM DnaA, and NapM at concentrations indicated in the figure legends. The reactions were performed at 37 °C for 30 min and stopped by placing on ice. Products were separated by TLC on a polyethylenemine cellulose strip (Merck) in 0.5 M LiCl and 1 M formic acid at room temperature for 45 min. The TLC plate was dried and autoradiographed.

### DNA synthesis assay in vitro

Following a previously published procedure^31^, the fractionation of DNA-replication activity in vitro was collected through ammonium sulfate. *M. tuberculosis* H37Ra was grown in 7H9 media for 7 days in roller bottles at 37 °C. The cells were harvested, washed, and resuspended with buffer A (25 mM HEPES/KOH (pH 7.6), 0.1 mM EDTA, 2 mM dithiothreitol, and 100 mM potassium glutamate). The supernatant was acquired by rupturing the cells with sonication and then precipitated slowly by the addition of ammonium sulfate at 4 °C. The obtained precipitate (fraction II) was resuspended and dialyzed for 60–90 min at 4 °C with buffer A. The protein concentration was estimated by Bio-Rad protein assay. In vitro DNA replication was carried out with a buffer including 40 mM HEPES-KOH (pH 7.6), 21.6 mM creatine phosphate (Fluka), 6 mM ATP, 500 µM each of GTP, CTP, and UTP, 100 µM each of dGTP, dCTP, and dTTP, 50 µM dATP, 50 µg ml^−1^ bovine serum albumin, 200 cpm M^−1^ of total deoxynucleotide α-^32^P dATP, 7% polyethylene glycol 10,000, 11 mM magnesium acetate, 35 µg creatine kinase (Sigma), and 2.5 µg supercoiled plasmid DNA (pUC-*oriC*). The reaction mixture was incubated at 30 °C for 90 min after adding 200 µg of fraction II. Total nucleotide incorporated within the supernatant was purified using a DNA purification kit (BioFlux, Malaysia), dotted on nylon membrane and measured by determining radioactivity. All the reactions were quantitated by Typhoon Variable Mode Imager and Image Quant software.

### Determination of DNA content by flow cytometry

DNA content was measured by flow cytometry as previously described with some modifications^32^. Cells were centrifugated at 1000 rpm for 3 min to remove clumps. Supernatant cells were centrifuged at a high speed and washed three times in phosphate-buffered saline (PBS). Then, the cells were fixed in 4% paraformaldehyde and 50 mM ammonium chloride. The fixed samples were permeabilized with 0.1% Triton X-100 and 2 mg ml^−1^ lysozyme^33^, and then stained with 100 μM 4,6-diamidino-2-phenylindole diluted in PBS for 20 min at room temperature. Stained cells were rinsed with PBS, then sonicated and passed through a 70 μm filter. Samples were gated based on forward and side scatter of unstained cells, and were analyzed using Beckman cytoFlex (USA) (Supplementary Fig. 9).

### Assays for mycobacterial growth and stress-inducible survival

To investigate the effect of *napM* or *dnaA* gene on the growth of *M. tuberculosis* H37Ra, pMV261-*napM*, pMV261-*dnaA*, and pMV261-*dnaA* -*napM* were produced and electroporated into the *M. tuberculosis* H37Ra strain. As many as 10^−5^–10^−6^ CFUs of recombinant mycobacterial strains were incubated in 5 ml Middlebrook 7H9 + 10% OADC + 0.05% Tween 80 + 0.2% glycerol medium for 14 days or were streaked on Middlebrook 7H10 + 10% OADC + 0.5% glycerol plates. The mycobacterial growth was measured as CFU from serial dilutions plated onto Middlebrook 7H10 + 10% OADC + 0.5% glycerol agar plates and incubated at 37 °C for 25 days. The wild-type and the *napM*-deleted *M. tuberculosis* strains were treated with the indicated concentrations of H_2_O_2_ or 0.05% SDS for a period of 24 h. Surviving cells were enumerated, and the data are expressed as survival percentage as compared to unexposed controls.

### Intracellular survival assay

The *Mus musculus* (mouse) monocyte/macrophages (RAW264.7) were maintained at 37 °C in a humidified incubator with 5% CO_2_ and infected with *M. tuberculosis* H37Ra at a multiplicity of infection of 3 as previously described^34^. Macrophages (RAW264.7) were incubated with a mutant or wild-type strain of *M. tuberculosis* H37Ra for 4 h, and the extracellular bacteria were washed away by replacing the culture supernatant with fresh medium. At the indicated times, RAW264.7 were lysed with 0.2% Triton X-100, and serial dilutions were plated on Middlebrook 7H10 solid medium. The *P* values of the relative growth data were calculated by unpaired two-tailed Student’s *t* test.

### Reporting summary

Further information on experimental design is available in the Nature Research Reporting Summary linked to this article.

## Supplementary information

Supplementary Information

Reporting Summary

## Data Availability

The datasets generated during the current study are available from the corresponding author on reasonable request.
