# Supplementary material for: NapM enhances the survival of Mycobacterium tuberculosis under stress and in macrophages
Source: Commun Biol. 2019 Feb 15;2:65. doi: 10.1038/s42003-019-0314-9 (PMC6377630; doi:10.1038/s42003-019-0314-9)
Supplement: Supplementary file 1 — Supplementary Information [file 42003_2019_314_MOESM1_ESM.pdf]

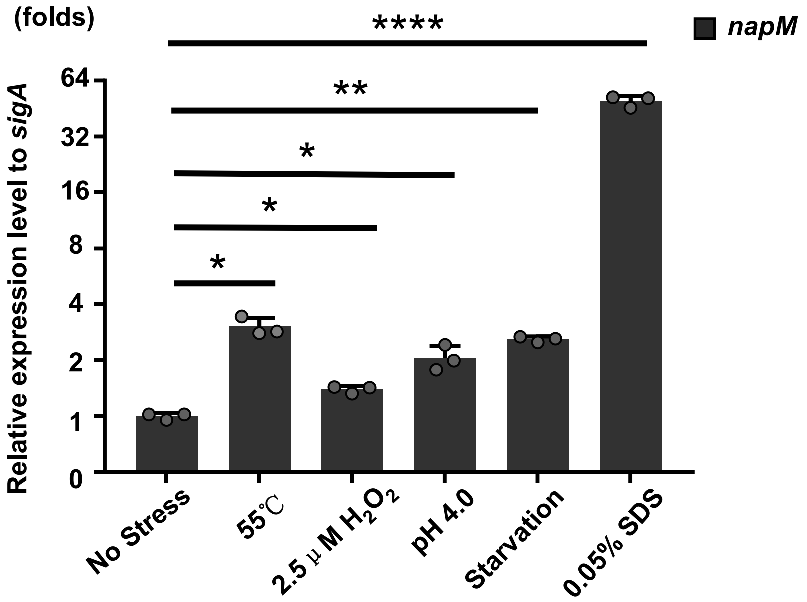

Supplementary Figure 1. qRT-PCR assays for *napM* expression in *M. tuberculosis* under several environmental stresses. The environmental stresses were oxidative stress, heat shock, acid shock, cell-membrance damage, and nutrition starvation. The results were normalized by invariant transcript *sigA* gene. Symbols represent each biological replicate and bars indicate means ± standard errors calculated from three independent experiments. Asterisk represents significant difference(**** p <0.0001, ** p <0.01, * p <0.05， two-tailed Student's *t*-test) between two groups.

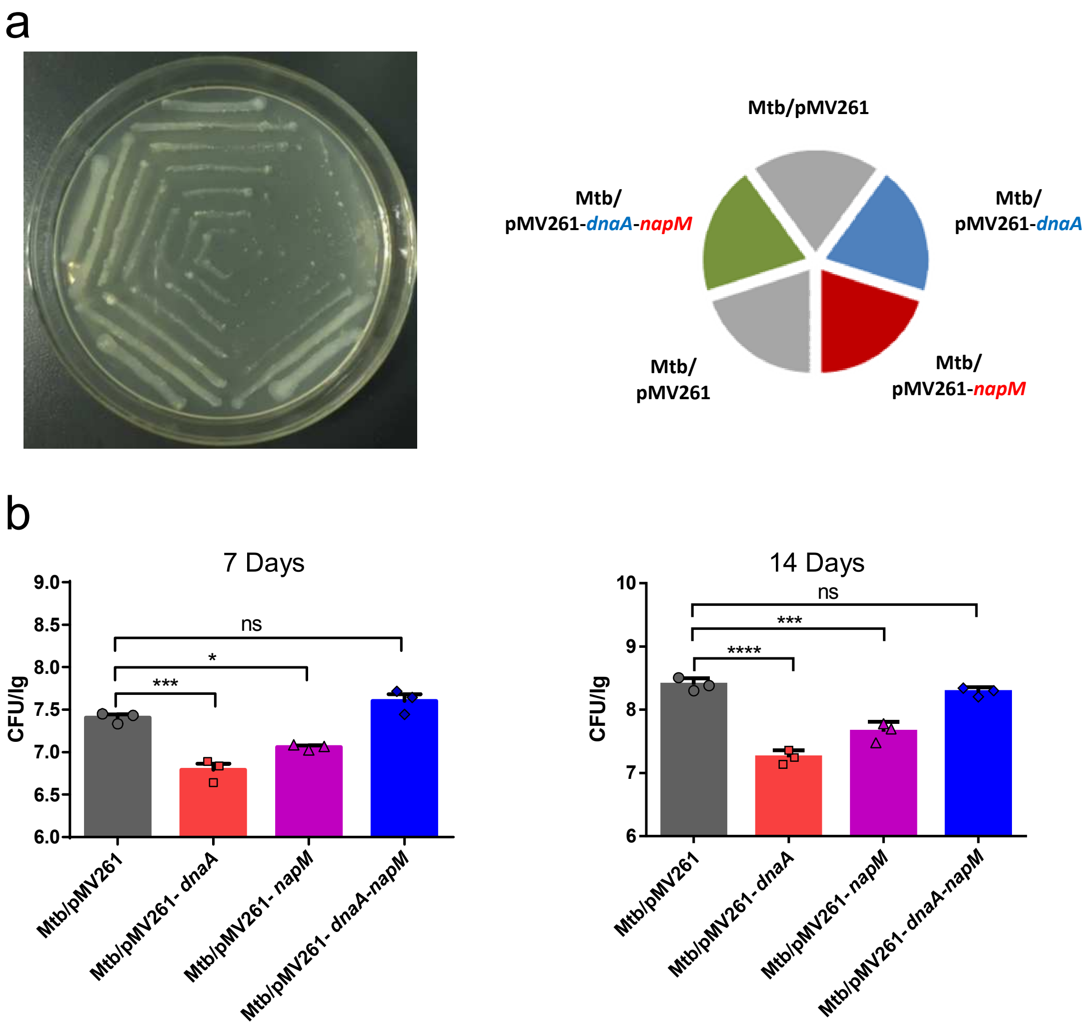

Supplementary Figure 2. Co-expression of *napM* and *dnaA* neutralized their respective inhibition on the growth of *M. tuberculosis* H37Ra. a. Recombinant mycobacterial strains were streaked on Middlebrook 7H10+10% OADC +0.5% glycerol agar plates and incubated at 37℃. *napM*-*dnaA* co-expression recombinant strain demonstrated similar growth as the wild-type strain containing an empty vector pMV261. **b.** Bacterial counts were assayed using liquid culture by determining colony-forming units (CFU) at two representative time points, 7 and 14 days. Symbols represent each biological replicate and bars indicate means ± standard errors calculated from three independent biological experiments. Asterisk represents significant difference(**** p <0.0001, *** p <0.001， * p <0.05， two-tailed Student’s *t*-test) between two groups.

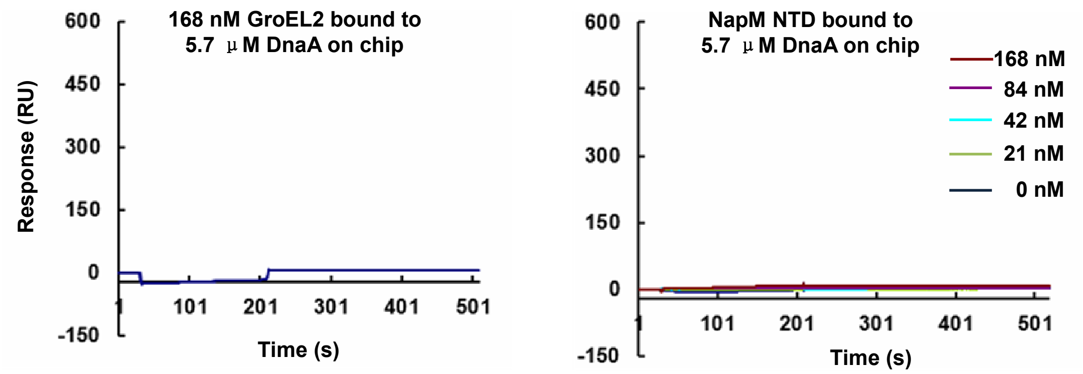

Supplementary Figure 3. Surface plasmon resonance (Biacore) analysis for the interaction between GroEL2/ NapM NTD and DnaA. 5.7 μM DnaA was immobilized on the CM5 sensor chip, and GroEL2 or NapM NTD was injected over the immobilized protein. Both protein showed no detectible binding to these substrates.

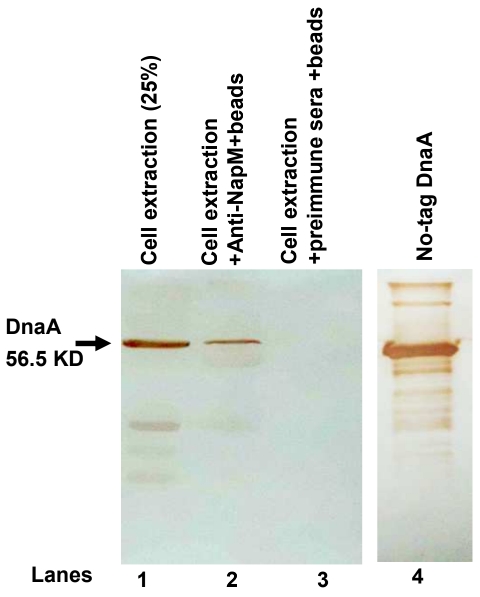

Supplementary Figure 4. Co-IP assays for the in vivo interaction between DnaA and NapM in *M. tuberculosis*. Exponentially growing cells of the recombinant *M. tuberculosis* containing DnaA-expression plasmid were harvested, resuspended and lysed. Rabbit anti-NapM serum was then added into the mixture. Protein A beads were conjugated with an antibody raised against NapM. The samples were analyzed by Western blot using mouse anti-DnaA antibody. DnaA was associated with NapM (lane 2), and no signal was detected for a negative control sample (lane 3).

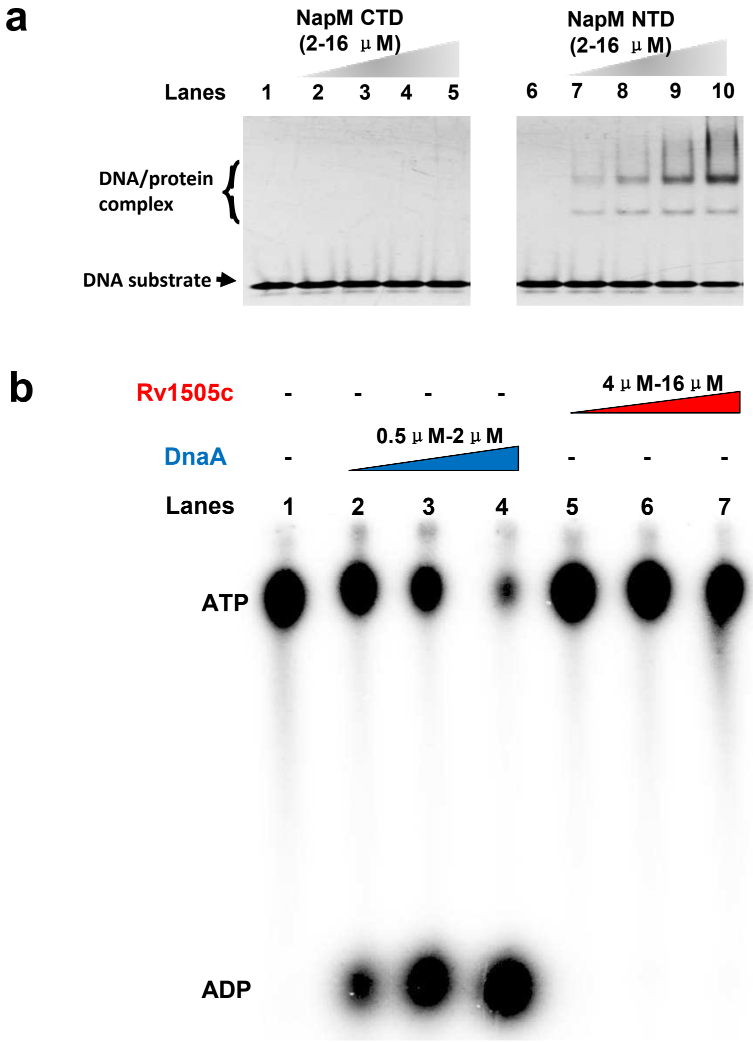

Supplementary Figure 5. a. Electrophoretic mobility shift assay for measuring the binding ability of NapM NTD and CTD regions to double-stranded DNA (30bp). DNA fragment was incubated with indicated amounts of C-terminal (left) or N-terminal (right) NapM proteins and analyzed on 5% polyacrylamide gel. **b.** TLC assays for the ATPase activity of DnaA and the negative control protein Rv1505c. A different concentrations of DnaA (0.5–2 μM) and Rv1505c (4–16 μM) was added into the reactions for [α-32P] ATP hydrolysis assay. The released phosphate was quantified using Typhoon Variable Mode Imager.

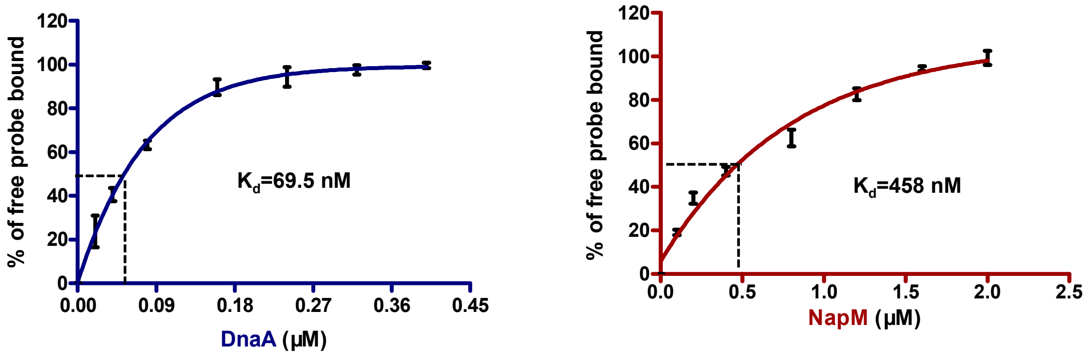

Supplementary Figure 6. Saturation curves of DnaA and NapM binding to *oriC*. Binding constants were determined by performing EMSA reactions with a 1 μM FITC-labeled *oriC* probe. These experiments were performed in triplicate and quantified using a Typhoon system (GE Healthcare, Fairfield, CA, USA). The percentage of bound probe was calculated by estimating the diminishing intensity of the free probe. Data were fitted with a sigmoidal curve, where 50% saturation corresponded with the K_d value. Error bars indicate standard error.

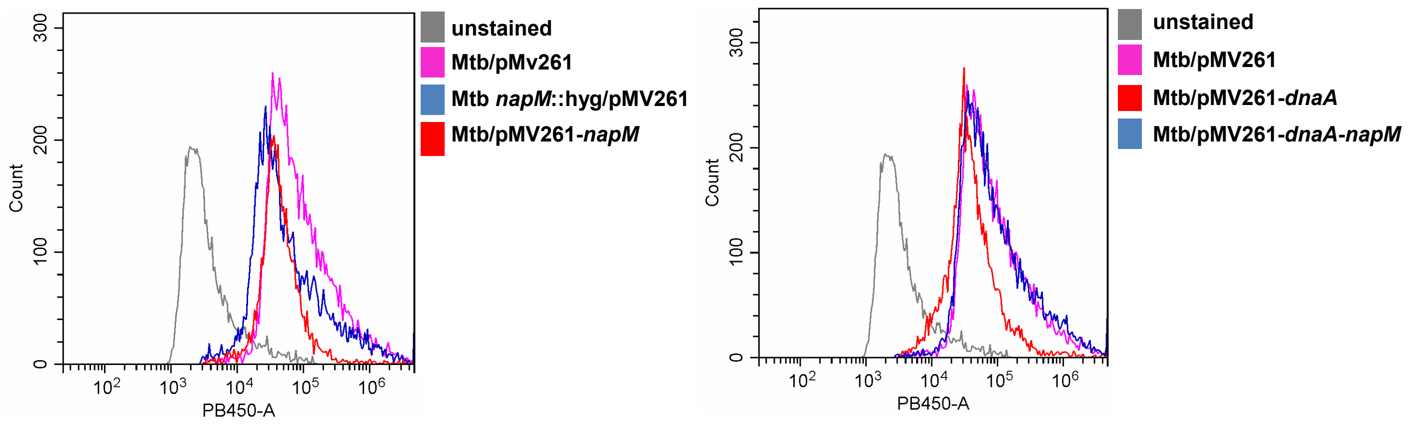

Supplementary Figure 7. Measurement of DNA content by flow cytometry. Representative flow cytometry histograms in the PB450-A channel, which measures DAPI fluorescence of unstained cells, wild type cells and the different genetic mutants. Histograms used to calculate DAPI mean fluorescence intensity (MFI) plotted for several biological replicates shown in Fig. 4b.

a

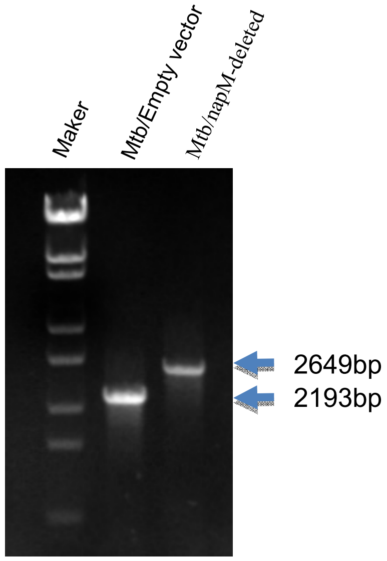

c

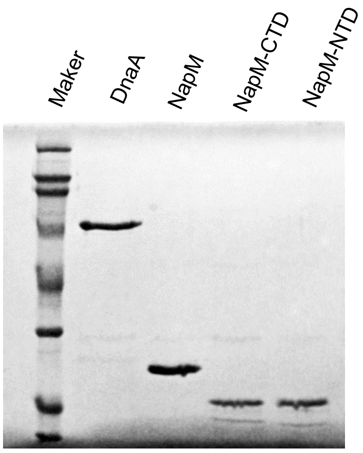

b

sequencing : Mtb/Empty vector (*napM*)
AGGTGACTCGATGCTGGAGCTCGCCATCCTGGGTCTGTTGATCGAGTCACCGATGCAT
GGCTACGAGTTGCGCAAGAGGCTGACCGGCCTGCTGGGCGCGTTTCGGGCATTTTCGT
ACGGTTCGTTATACCCGGCGCTGCGACGCATGCAGGCCGACGGGTTGATCGCCGAGAA
TGCCGCCCCAGCGGGCACCCCGGTGCGGCGAGCACGACGCGTTTACCAACTGACCGAC
AAGGGTCGCCGTCGATTCGGCGAGCTGGTGGCCGACACCGGCCCACACAACTACACC
GACGACGGTTTCGGGGTGCACCTGGCGTTCTTCAACCGCACCCCGGCCGAAGCGCGGA
TGCGCATCCTGGAAGGCCGGCGCCGCCAGGTCGAGGAACGCCGGGAAGGCTTGCGCG
AGGCGGTGGCGCGGGCCAGCAGTTCCTTCGACCGCTACACCCGCCAATTGCATCAACT
CGGGCTCGAGTCCAGCGAGCGCGAGGTCAAGTGGCTCAACGAGCTCATCGCCGCGGA
ACGGGCAGCACCCAACCCCGCCGAACAGACGTAAACCCAGCACG

sequencing : Mtb/napM-deleted (hyg)
AGGTGACTCGGTGACACAAGAATCCCTGTTACTTCTCGACCGTATTGATTCGGATGAT
TCCTACGCGAGCCTGCGGAACGACCAGGAATTCTGGGAGCCGCTGGCCCGCCGAG
CCCTGGAGGAGCTCGGGCTGCCGGTGCCGCCGGTGCTGCGGGTGCCCGGCGAGA
GCACCAACCCCGTACTGGTCGGCGAGCCCGGCCCGGTGATCAAGCTGTTCGGCGA
GCACTGGTGCGGTCCGGAGAGCCTCGCGTCGGAGTCGGAGGCGTACGCGGTCCTG
GCGGACGCCCCGGTGCCGGTGCCCCGCCTCCTCGGCCGCGGCGAGCTGCGGCCC
GGCACCGGAGCCTGGCCGTGGCCCTACCTGGTGATGAGCCGGATGACCGGCACCA
CCTGGCGGTCCGCGATGGACGGCACGACCGACCGGAACGCGCTGCTCGCCCTGGC
CCGCGAACTCGGCCGGGTGCTCGGCCGGCTGCACAGGGTGCCGCTGACCGGGAAC
ACCGTGCTCACCCCCCATTCCGAGGTCTTCCCGGAACTGCTGCGGGAACGCCGCGC
GGCGACCGTCGAGGACCACCGCGGGTGGGGCTACCTCTCGCCCCGGCTGCTGGAC
CGCCTGGAGGACTGGCTGCCGGACGTGGACACGCTGCTGGCCGGCCGCGAACCCC
GGTTCGTCCACGGCGACCTGCACGGGACCAACATCTTCGTGGACCTGGCCGCGACC
GAGGTCACCGGGATCGTCGACTTCACCGACGTCTATGCGGGAGACTCCCGCTACAG
CCTGGTGCAACTGCATCTCAACGCCTTCCGGGGCGACCGCGAGATCCTGGCCGCGC
TGCTCGACGGGGCGCAGTGGAAGCGGACCGAGGACTTCGCCCGCGAACTGCTCGC
CTTCACCTTCCTGCACGACTTCGAGGTGTTCGAGGAGACCCCGCTGGATCTCTCCG
GCTTCACCGATCCGGAGGAACTGGCGCAGTTCCTCTGGGGGCCGCCAGACACCGC
CCCCGGCGCCTAAACCCAGCACG

Supplementary Figure 8. a. Specific primers were designed corresponding to the upstream and downstream sequence of the gene, and PCR amplification was performed using the genomes of wild-type and knockout mutants. The fragment length of the PCR product is different, indicating that *napM* may be exchanged with hyg. **b.** Sequencing analysis of the PCR product. The sequence was exactly consistent with *napM* in the wild type but hyg in the knockout mutants. **c.** The purity of these proteins (DnaA, NapM, NapM CTD, NapM NTD) was estimated by SDS-PAGE.

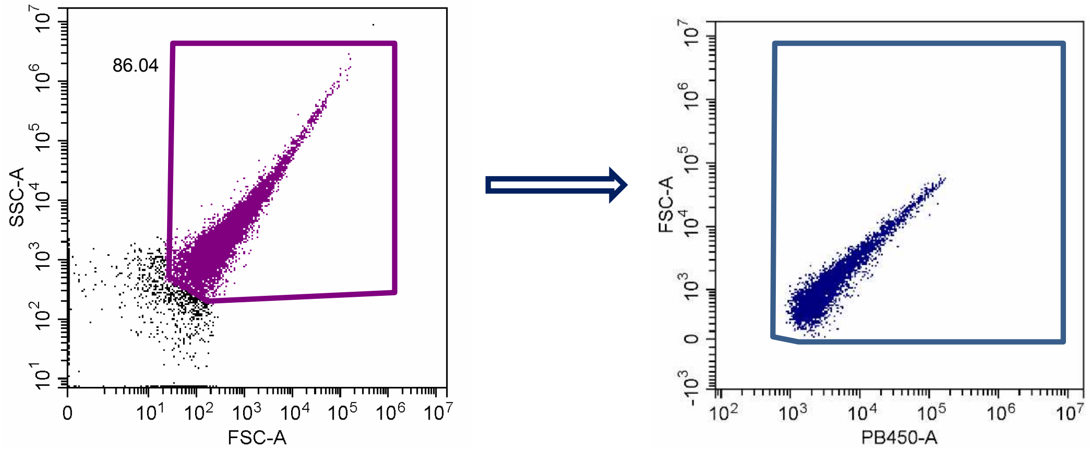

Supplementary Figure 9. Gating strategy. DNA content was measured by flow cytometry using purely cultured *Mycobacterium tuberculosis* H37Ra. Representative flow cytometric gating with unstained cells was used to identify cell populations and fluorescence intensity of samples.

Supplementary Table 1. the expression of NapM in mycobacteria is induced by stress environment

fold change	stress	data source
3.5	capreomycin	Fu, L.M. and Tai, S.C., 2009
2.8	PA-824	Fu, L.M. and Tai, S.C., 2009
2.3	meropenem	Lun *et al*., 2014
2.5	HIV-blood	Ryndak *et al*., 2014
3.62	Arachidonic acid	TB database*
2.6	Capreomycin	
2.76	Cumene Hydroperoxide + Arachidonic acid	
1.63	Defined medium	
1.85	Defined medium + Oleic acid	
3.45	Defined medium + Palmitic acid	
6.26	Drug #121940	
-4.24	Hypoxia	
3.11	Linoleic acid	
2.15	Oleic acid	
1.88	Palmitic acid	
2.89	Palmitic acid + Arachidonic acid	
3.27	Palmitic acid + Oleic acid	
4.63	Procept 6778	
1.78	Pyrazinamide	
5.66	Roxithromycin	

*For a gene to be listed, it must satisfy the criteria of p-value ≤0.01 and significance>1. The data from most acute or long-lasting stress treatment were extracted .
